# Severe leptospirosis and pancreatitis; A case series from a leptospirosis outbreak in Anuradhapura district, Sri Lanka

**DOI:** 10.1186/s12879-016-2010-4

**Published:** 2016-11-07

**Authors:** N. J. Herath, C. J. Kamburapola, S. B. Agampodi

**Affiliations:** 1 Teaching Hospital, Anuradhapura, Sri Lanka; 2 Department of Community Medicine, Faculty of Medicine and Allied Sciences, Rajarata University of Sri Lanka, Saliyapura, 50008 Sri Lanka

## Abstract

**Background:**

Acute pancreatitis is identified as an atypical and rare presentation of leptospirosis. In this report, we discuss a case series of severe leptospirosis with pancreatitis as a main complication during an unusual outbreak of leptospirosis in Anuradhapura district, Sri Lanka.

**Case presentation:**

We retrospectively reviewed clinical presentation, investigations, treatment and outcome of six confirmed cases of severe leptospirosis admitted to intensive care unit (ICU) of Teaching Hospital, Anuradhapura, within a three months period from November 2014 to January 2015.

All six patients were previously healthy paddy farmers presented with fever, myalgia and arthralgia. Four patients had abdominal pain, nausea and vomiting on admission. Hypotension, Neutrophilic leukocytosis and thrombocytopaenia was detected in all patients in the initial stage. Four patients had serum amylase more than 900 (range 941–2420). All patients had acute kidney injury and hepatitis. Significantly elevated amylase and low serum calcium were present in 4 cases. Five patients recovered without any evidence of residual organ damage, but one succumbed to the illness.

**Conclusion:**

This case series emphasizes the importance of identification of acute pancreatitis as a common complication of Leptospirosis, in order to reduce mortality and morbidity.

## Background

Leptospirosis is a poverty related, neglected tropical disease. Increased frequency of leptospirosis outbreaks were noted in Caribbean islands, Central and South Americas and South East Asia recently [1]. The disease is caused by a spirochete transmitted by direct or indirect contact with the urine of an infected animal through abrasions of the skin, or mucous membranes of mouth, nose, ear or conjunctiva. It is considered as the most widespread zoonotic disease [2] affecting all rodent, mammals, birds, marsupials and amphibians. Over 250 serovars, classified into more than 31 serogroups are reported from tropics and subtropics [3]. DNA hybridization based genetic classification shows at least 21 species of *Leptospira*.

Only a minor percentage of leptospirosis infections are symptomatic. Among symptomatic patients, initial leptospireamic phase is febrile with clinical features such as fever, arthralgia, myalgia, pharyngitis, cough, headache, chills, rigors, conjunctival suffusion and non pruritic rash [3]. Resolution of the febrile phase occurs with the clearance of Leptospira from bloodstream. The immune phase can occur in less than 10 % of patients, and was first described as Weil’s disease more than a century ago [4]. In this phase, fever, jaundice, hepatitis, acute kidney injury, myocarditis, hemorrhage and rarely pancreatitis can occur. Virtually any organ system can be involved during this phase [5]. Even though the classical Weil’s disease is considered “pathognomic” for leptospirosis by some clinicians, increasing number of atypical presentations are observed among leptospirosis recently. In Sri Lanka, differences of clinical presentations and sequale was described previously and attributed to micro geographic changes [6]. In this paper, we describe a series of patients with severe leptospirosis, presented to a single hospital within a period of three months, during an outbreak of leptospirosis.

## Case presentation

We reviewed six cases of severe leptospirosis admitted to Intensive Care Unit (ICU) of Teaching Hospital, Anuradhapura, Sri Lanka within a three months period from November 2014 to January 2015. Data on socio-demographic factors, presenting clinical features, examination findings, investigations and management was collected through direct interviews during follow up clinic visits and from hospital records. Leptospirosis was confirmed in all patients with microscopic agglutination test (MAT) for acute sera, using a panel of 12 serovars in Medical Research Institute (MRI), Sri Lanka (National reference laboratory). Five patients were reviewed in outpatient clinics at two weeks after being discharged from the hospital. Informed written consent was taken from all the patients recruited for the study.

All six patients were previously healthy, male paddy farmers from Anuradhapura district. They all had fever, myalgia and arthralgia on presentation. Four patients (case 1–4) complained of abdominal pain radiating to back and nausea and vomiting on admission (Table 1).

**Table 1:** Presenting clinical symptoms of six confirmed cases of leptospirosis complicated with pancreatitis

Case number	Age	Fever	Myalgia	Chest pain	Abdominal pain
01	57	+	+	-	+
02	48	+	+	-	+
03	28	+	+	-	+
04	47	+	+	-	+
05	33	+	+	-	-
06	44	+	+	-	-

On examination, four patients had conjunctival suffusion and three were icteric (Table 2). None of the patients were edematous and only a single patient had bleeding manifestations. Epigastric tenderness and guarding (tensing of the abdominal wall muscles to guard inflamed organs within the abdomen from the pain of pressure upon them) was elicited with four patients who were having abdominal pain on admission. Hypotension (systolic blood pressure < 90 mm Hg) was detected in all cases.

**Table 2:** Clinical findings of six confirmed cases of leptospirosis

Case number	General examination					Cardiovascular System		Respiratory system		Abdomen	
	Conjunctival suffusion	Pallor	Icterus	Edema	Bleeding manifestations	Tachycardia (PR > 90/min)	Low BP (SBP < 90 mmHg)	Tachypnoea (RR > 20)	Crepitations	Tenderness	Hepatomegaly
01	+	-	+	-	-	+	+	-	+	+	-
02	-	+	+	-	-	-	+	-	+	+	+
03	+	-	-	-	-	+	+	+	+	+	-
04	+	-	-	-	-	+	+	+	+	+	-
05	-	-	-	-	-	-	+	-	+	-	+
06	+	-	+	-	+	+	+	+	+	-	+

*PR* Pulse rate, *BP* Blood pressure, *SBP* Systolic blood pressure, *RR* Respiratory rate

Neutrophilic leukocytosis (White Blood Cell count (WBC) > 11,000/ μl) and thrombocytopaenia (Platelet count <100,000/μl) was detected in all patients in the initial stage. All patients had acute kidney injury (AKI) with varying severity with proteinuria and microscopic haematuria. Liver enzymes (6/6), direct hyper-bilirubinemia (5/6) and high alkaline phosphatase (ALP) (in 6/6) had been present in all cases at the presentation. Significantly elevated amylase (> five times the upper normal value), and low serum calcium levels were present in cases 1-4 (Tables 3 and 4). There were marked epigastria tenderness and guarding in cases 1–4. Serial analysis of serum amylase showed initial rapid rise even before establishment of AKI. Thereafter, serum amylase level plateaued followed by declining to normal. Low serum calcium supported the diagnosis. We could not perform CT in view of AKI, but this would have been supportive evidence for diagnosis of acute pancreatitis.

**Table 3:** Blood and urine biochemistry of six confirmed cases of leptospirosis

Case	FBC					RFT		UFR			SE (mEq/L)			ABG	
Number	WBC (x10^9^/L)	N%	PCV (%)	Hb (g/dl)	Plt (x10^9^/L)	Cr (mmol/l)	BUN (mmol/l)	Albumin	leucocytes/field	RBC/filed	Na	K	Ca	pH	HCO3-
1	13.5	87.4	34	10.9	23	177→379	16.1	2+	10–20	30–40	141	5	2.02	7.35	18.6
2	13.7	85.8	35.5	11.7	11	281→448	20.8	2+	15–20	20–30	129	5	1.50	7.32	12.8
3	18.2	86.2	36.9	11.6	13	429→464	37.5	2+	10–15	40–50	140	4	2.05	7.36	12.8
4	15.1	78	35.4	11.6	56	247→269	13	2+	10–15	20–30	140	2.9	2.00	7.38	14
5	14.4	82	36.5	12.6	27	144→167	13.3	2+	10–15	30–40	143	3.2	2.06	7.44	18.4
6	19	93	32	10.3	11	306→434	14	2+	15–20	40–50	139	4.5	1.76	7.4	18.4

*FBC* full blood count, *WBC* White blood cell count, *PCV* Packed cell volume, *Hb* Haemoglobin, *Plt* Platelet count, *RFT* Renal function tests, *Cr* Creatinine, *BUN* Blood uria nitrogen, *UFR* Urine full report, *SE* serum electrolytes, *Na* Sodium, *K* Potassium, *Ca* Calcium, *ABG* Arterial blood gasses

**Table 4:** Blood and urine biochemistry of six confirmed cases of leptospirosis

Case No	LFT							
	AST (units/l)	ALT (units/l)	Total Bilirubin (μmol/L)	Direst bilirubin (μmol/L)	INR	ALB (g/L)	Serum Amylase (units/l)	ALP units/l)
1	96	66	20.3	14.6	1.1	39	983→375	266
2	109	99	105	-	1.34	33	2162→ 2420	254
3	78	38	194	184	1.27	30	2365→204	292
4	148	148	53.5	29.1	1.15	32	941→285	270
5	85	77	54	32.8	NA	30	107	260
6	132	116	100	86.9	1.08	33	193	280

*AST* Aspartate aminotransferase, *ALT* Alanine aminotransferase, *INR* International normalized ratio, *ALB* Albumin, *ALP* Alkaline phosphatase

All patients had multiple complications (Table 5). They were treated in medical ICU and needed two or more inotropes. All of them were given IV C penicillin, cefotaxime and doxycycline for 10 days. Low dose IV hydrocortisone was given initially to all patients to improve blood pressure. Case number 2 and 6 needed dialysis and in others kidney functions improved without renal replacement therapy.

**Table 5:** Complications and clinical outcome among six confirmed cases of leptospirosis

Case No.	Complications	Outcome
01	Pancreatitis, AKI, hepatitis	Fully recovered
02	Pancreatitis, AKI, hepatitis,	Died
03	Pancreatitis, AKI, hepatitis, myocarditis	Fully recovered
04	Pancreatitis, AKI, hepatitis, myocarditis	Fully recovered
05	Myocarditis, AKI, hepatitis	Fully recovered
06	AKI, hepatitis, myocarditis	Fully recovered

Case number 2 died during acute illness (on day 7) due to refractory hypotension and multiorgan failure. Rests of the patients were reviewed after 2 to 4 weeks post discharge. They all had complete recovery, without any evidence of residual organ damage.

## Discussion

Acute pancreatitis was previously reported as an uncommon complication of leptospirosis [7–9]. In Sri Lanka, pancreatitis was previously reported in one confirmed [10] and one probable [11] cases of leptospirosis.

The exact mechanism of acute pancreatitis in leptospirosis is not fully described. An immunological basis for pathogenesis of leptospirosis including Toll like receptor (TLR) 2 activation is described recently [12]. The most consistent pathologic finding in leptospirosis is vasculitis of capillaries manifested by endothelial oedema, necrosis and lymphocytic infiltration. Small vessel vasculitis and ischemic injury leading to activation of proteolytic enzymes and auto-digestion is a possible mechanism [12]. All the patients described in the current series, were hemodynamicaly unstable at presentation or within the first 24 h of hospital stay. Refractory hypotension can lead to multi-organ failure which could be another cause for acute pancreatitis in these patients.

Whatever the mechanism behind, finding of pancreatitis among four out of six confirmed cases admitted to ICU during a single epidemic of leptospirosis in a geographically defined region needs further investigation. In another study done during this same outbreak of leptospirosis in Anuradhapura district, but in a different hospital, clinical presentations were reported as different from 2011 outbreak [13]. This observation could be explained by previous observations of micro geographical changes of leptospirosis in Sri Lanka and the hypothesis on etiological differences of epidemic and endemic leptospirosis [6]. Alternatively, the observed outbreak of pancreatitis could be due to a new strain or a slight difference in the endemic strain of *Leptospira* as described previously [14]. We have not carried out molecular studies during this outbreak to evaluate latter hypothesis. However, we propose further studies to explain different clinical manifestations by different strains of *Leptospira* within the same localities during different outbreaks.

There are reports of co-infections of *Leptospira* and Hantavirus infections in Sri Lanka [15]. Similarly co-infections could occur in these patients with various other infectious causes of acute pancreatitis i.e. parasites (Ascaris, toxoplasma), viruses (hepatits B, Coxsackie, CMV, mumps), bacteria (Mycoplasma, Salmonella). We have not excluded other co-infections in the present case series, which could be a rare alternative explanation for the observation.

## Conclusion

This case series highlight the importance of identification of acute pancreatitis as a complication of leptospirosis. Awareness regarding this important complication is important to decrease risk of lethal outcome.
